# Butyrate Alleviates Cytokine-Induced Barrier Dysfunction by Modifying Claudin-2 Levels

**DOI:** 10.3390/biology10030205

**Published:** 2021-03-09

**Authors:** Xinyi Huang, Tadayuki Oshima, Toshihiko Tomita, Hirokazu Fukui, Hiroto Miwa

**Affiliations:** Division of Gastroenterology and Hepatology, Department of Internal Medicine, Hyogo College of Medicine, Nishinomiya 663-8501, Japan; ds1902@hyo-med.ac.jp (X.H.); tomita@hyo-med.ac.jp (T.T.); hfukui@hyo-med.ac.jp (H.F.); miwahgi@hyo-med.ac.jp (H.M.)

**Keywords:** short-chain fatty acids, butyrate, claudin-2, intestinal barrier disruption

## Abstract

**Simple Summary:**

The influence of Short-chain fatty acids (SCFAs) on barrier function under pathological conditions has not been assessed, and the regulation of the tight junction (TJ) proteins by SCFAs under pathological conditions has not been fully elucidated. We therefore aimed to evaluate the effect of SCFAs on intestinal barrier function under cytokine-stimulated conditions. Butyrate, but not acetate, propionate, or succinate, ameliorated the tumor necrosis factor-alpha (TNF-α)/interferon-gamma (IFN-γ)-induced decrease in transepithelial electrical resistance (TEER). TNF-α/IFN-γ stimulation significantly increased the protein level of claudin-2 and decreased the level of claudin-3. Butyrate significantly attenuated the upregulation of claudin-2 induced by TNF-α/IFN-γ. Similarly, butyrate blocked the decrease in TEER and the upregulation of claudin-2 induced by interleukin-13, without changing the level of other TJ proteins. Our results suggested that butyrate is the main component of SCFAs to alleviate barrier dysfunction and that claudin-2 is the major target of this SCFA. It is hoped that these results will facilitate the development of treatments for diseases related to intestinal barrier impairment.

**Abstract:**

Gastrointestinal (GI) disorders such as celiac disease and inflammatory bowel disease are attributed to intestinal barrier disruption. Imbalance of cytokines has been reported in the intestinal epithelium of patients with GI disorders. Short-chain fatty acids (SCFAs), derived from the fermentation of dietary fiber in the intestine, have been reported to benefit the intestinal barrier. Accordingly, we evaluated the effect of specific SCFAs on intestinal barrier function under cytokine-stimulated conditions. Caco-2 cells were cultured on insert membranes to generate monolayers, which then were used to investigate the effects of SCFAs. Tumor necrosis factor-alpha (TNF-α), interferon-gamma (IFN-γ), or interleukin-13 (IL-13) was added to the basolateral side of the membrane while SCFAs were added to the apical side. After a 24 h stimulation, transepithelial electrical resistance (TEER) was measured, and the protein levels of claudin-1, claudin-2, claudin-3, claudin-4, occludin, and zonula occludens-1 (ZO-1) were evaluated by Western blot. Butyrate, but not acetate, propionate, or succinate, ameliorated the TNF-α/IFN-γ-induced decrease in TEER. TNF-α/IFN-γ stimulation significantly increased the protein level of claudin-2 and decreased the level of claudin-3. Butyrate significantly attenuated the upregulation of claudin-2 induced by TNF-α/IFN-γ. Butyrate blocked the decrease in TEER and the upregulation of claudin-2 induced by IL-13 without changing the level of other tight junction proteins. Our results suggested that butyrate is the main component of SCFAs to alleviate barrier dysfunction and that claudin-2 is the major target of this SCFA.

## 1. Introduction

Irritable bowel syndrome (IBS), inflammatory bowel disease (IBD), and celiac disease are chronic gastrointestinal (GI) diseases characterized by inflammation and injury of the intestinal barrier [1,2]. The levels of pro-inflammatory and anti-inflammatory cytokines in the peripheral circulatory system and intestinal mucosa have been reported to be elevated in patients with IBS and IBD [3,4]. One of the pro-inflammatory cytokines, tumor necrosis factor-alpha (TNF-α), has been well studied and shown to be upregulated in IBS [5]. Interferon-gamma (IFN-γ) is also an important effector cytokine in IBS [6]. Levels of interleukin-13 (IL-13) have been shown to correlate with exacerbation and pathophysiology of IBD [7]. Therefore, dysregulation of cytokines is closely associated with intestinal barrier disruption.

The tight junction (TJ), which is generated by the assembly of several proteins, including claudins, occludin, and zonula occludens (ZO), is a complex formed between neighboring epithelial cells; TJs play a critical role in controlling the permeability of the paracellular transport pathway [1]. Claudins form a large family of TJ-associated transmembrane proteins that define the pore pathway, which is a high-capacity, size- and charge-selective paracellular route, while ZO-1 and occludin are implicated in the leak pathway, which is a low-capacity, paracellular route that does not discriminate between solutes on the basis of charge, and allows limited flux of large molecules [8]. It is important for the intestinal epithelium to maintain gastrointestinal homeostasis to protect against invasion of the body by pathogens. Furthermore, GI diseases such as IBS are associated with barrier dysfunction caused by impaired TJs [1].

Short-chain fatty acids (SCFAs), which are derived from the fermentation of dietary fiber in the colon, are saturated fatty acids with a chain length ranging from one to six carbon atoms [9]. Different SCFAs are produced by different microbes, and the proportions of individual SCFAs vary dynamically depending on the site within the colon and specific intestinal microenvironment [10]. Some studies have shown that SCFAs benefit the gut, probably by activating pathways that mediate protective immunity and decrease GI tissue inflammation [11], improving intestinal barrier function, reducing inflammation and oxidative stress [12], and promoting the reassembly of TJs [13]. However, different SCFAs have diverse effects on the GI tract, and not all SCFAs have protective effects on the gut [14,15]. Among the most common SCFAs, butyrate has been widely studied for its effect on the intestinal barrier [13,16]. Nonetheless, the influence of each SCFA on barrier function under pathological conditions has not been assessed, and the regulation of TJ proteins by SCFAs under pathological conditions has not been fully elucidated.

In the present study, we assessed the effects of different SCFAs and investigated the targets of various SCFAs in the context of the intestinal barrier under the condition of inflammation.

## 2. Materials and Methods

### 2.1. Reagents and Antibodies

Sodium acetate (CAS127-09-3), sodium propionate (CAS137-40-6), sodium butyrate (CAS156-54-7), and disodium succinate (CAS150-90-3) were purchased from Wako (Tokyo, Japan). Recombinant human TNF-alpha (210-TA), IFN-γ (285-IF), and recombinant human IL-13 (213-ILB) were purchased from R&D Systems, Inc. (Minneapolis, MN, USA). Antibodies against claudin-1 (51-9000), claudin-2 (32-5600), claudin-3 (34-1700), claudin-4 (32-9400), occludin (33-1500), ZO-1 (61-7300 and 33-9100), Alexa 488-conjugated anti-mouse IgG antibody (A11001), and 4′,6-diamidino-2-phenylindole (D3571) were obtained from Thermo Fisher Scientific, Inc. (Waltham, MA, USA). Anti-glyceraldehyde phosphate dehydrogenase (GAPDH) antibody was obtained from Cell Signaling Technology, Inc. (Danvers, MA, USA). Mounting solution (S3023) was from Dako North America, Inc. (Carpinteria, CA, USA).

### 2.2. Cell Culture

Caco-2, a colonic epithelial cell line derived from a colorectal adenocarcinoma, was purchased from the American Type Culture Collection (ATCC; Rockville, MD, USA) and used to generate monolayers. Following seeding in Transwell^TM^-inserts (passage 60 to 80, 200 µL containing 1 × 10^5^ cells/well), cells were cultured with Dulbecco’s modified Eagle’s medium and supplemented with 10% fetal bovine serum, 1× penicillin–streptomycin, and 1× MEM non-essential amino acid solution in 37 °C, 5% CO_2_ incubator. The medium was replaced every 2 days.

### 2.3. Measurement of Transepithelial Electrical Resistance (TEER)

Electrical resistance across the Caco-2 epithelial monolayer was measured using a MILLICELL-ERS^®^ instrument (Millipore Corporation, Bedford, MA, USA) with “chopstick” electrodes; TEER was calculated as reported previously [17]. In brief, the values obtained from blank inserts were subtracted to give the net resistance, which was multiplied by the membrane area to give the resistance in area-corrected units (Ω·cm^2^). The change in electrical resistance was represented by percentage baseline resistance, which was calculated as follows: %baseline resistance = ((resistance from each time point) − (resistance from a blank insert))/((baseline resistance) − (resistance from a blank insert)) × 100, where baseline resistance was the resistance at time point 0 [18].

### 2.4. Treatment of Monolayers

Experiments were performed 4 days after seeding in insets. A combination of TNF-α and IFN-γ (10 ng/mL) [19,20]) or IL-13 (10 ng/mL) was added to the medium on the basolateral side of the insert while SCFAs (1, 2, 4 mM) [21] were added to the medium on the apical side. TEER tests or harvesting of cells for protein extraction were performed after 24 h of stimulation. Extracted proteins were then used for Western blotting analysis.

### 2.5. Western Blotting

Total protein was extracted by resuspension of the cell in reduced lysis buffer (0.5 M Tris-hydrochloride (HCL) (pH 6.8), 10% glycerol, 10% sodium dodecyl sulfate (SDS), 1 tab of protein inhibitor) [22]. Protein quantification was conducted with the DC^TM^ protein assay (Bio-Rad, Hercules, CA, USA). The procedure was conducted in line with the user manual of the DC^TM^ protein assay kit. Sodium dodecyl sulfate–polyacrylamide gel electrophoresis (SDS–PAGE) and Western blotting were performed after quantification. Polyvinylidene difluoride (PVDF) membranes with transferred protein were blocked by 5% skim milk/0.1% Tris-buffered saline–Tween (TBST). The membrane was incubated with primary antibodies of claudin-1, claudin-2, claudin-3, claudin-4, occludin, and ZO-1 at 4 °C overnight. Membranes were then washed and incubated with secondary horseradish peroxidase-conjugated anti-rabbit or anti-mouse IgG antibody for 1 h at room temperature. After washing, the ECL Select or Prime Western Blotting Detection Reagent (GE Healthcare, Chicago, UK) was used, and a chemiluminescent signal was detected using an ImageQuant LAS 500 instrument (GE Healthcare). Bands were then analyzed using ImageJ software (National Institutes of Health, Bethesda, MD, USA). GAPDH, a housekeeping protein, was probed to permit the normalization of protein loading among different lanes. Relative quantities were calculated as fold changes compared to the corresponding control.

### 2.6. Epithelial Permeability

Fluorescein isothiocyanate-labeled dextran (FITC-dextran; FD4, Sigma, St. Louis, MO, USA) was used as a permeable tracer [18]. Caco-2 epithelial monolayers were stimulated with TNF-α/IFN-γ (10 ng/mL) or IL-13 (10 ng/mL) and butyrate (2 mM) for 24 h. Hanks’ Balanced Salt Solution (HBSS) containing 10 mg/mL FITC-dextran (FD4) was added to the apical side of the Transwell^TM^-inserts. After 1 h incubation, HBSS from the basolateral side was collected, and the absorbance of FITC-dextran was determined at an excitation wavelength of 485 nm and an emission wavelength of 530 nm using a microplate fluorometer (Infinite M200, Tecan, Männedorf, Switzerland). Data were expressed as a percentage of control.

### 2.7. Immunofluorescence Staining

Caco-2 cells were seeded onto coverslips and exposed to TNF-α/IFN-γ (10 ng/mL) or IL-13 (10 ng/mL) with or without butyrate after confluence. Methanol (10 min) was used for monolayer fixation, and ZO-1 was stained. Primary mouse anti-ZO-1 antibody (1:100) and secondary antibody (1:1000) were used. The nucleus was stained by 4′,6-diamidino-2-phenylindole. After mounting with fluorescence mounting medium, samples were observed, and pictures were captured by a fluorescence upright microscope (BX53; Olympus, Tokyo, Japan).

### 2.8. Statistical Analysis

Representative data are presented as mean ± SD from at least three experimental replicates. Data were analyzed using two-tailed one-way ANOVA followed by Sidak’s multiple comparisons tests, if appropriate. Analyses were performed using Prism 6 (GraphPad Software, Inc., La Jolla, CA, USA). Differences with *p* < 0.05 were regarded as statistically significant.

## 3. Results

### 3.1. TNF-α/IFN-γ Induces Barrier Dysfunction and Regulates the Levels of TJ Proteins

**Figure 1:**
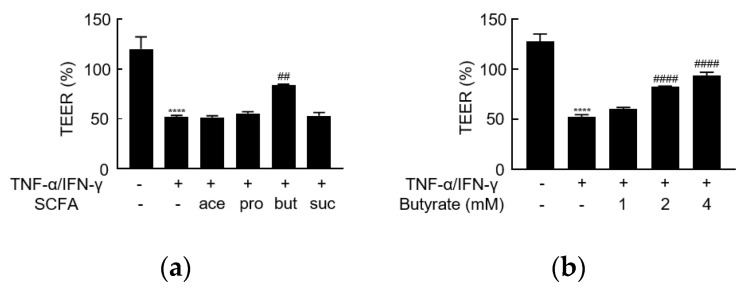
The effect of short-chain fatty acids (SCFAs) on the TNF-α/IFN-γ-induced barrier dysfunction. (**a**) Transepithelial electrical resistance (TEER) values were assessed and are presented as a percentage of control (time point 0), following stimulation on the basolateral side of the membrane with the combination of TNF-α/IFN-γ (10 ng/mL), and on the apical side with acetate (ace), propionate (pro), butyrate (but), or succinate (suc) (2 mM). (**b**) The concentration-dependent effect of butyrate on the TNF-α/IFN-γ-induced barrier dysfunction. **** *p* < 0.0001 compared to medium-treated control; ^##^ *p* < 0.01 compared to the TNF-α/IFN-γ group; ^####^ *p* < 0.0001 compared to TNF-α/IFN-γ group.

After 4 days of culturing on the insert membranes, Caco-2 monolayers were exposed to TNF-α/IFN-γ on the basolateral side for 24 h. TNF-α/IFN-γ significantly decreased TEER (Figure 1a), suggesting that TNF-α/IFN-γ exposure disrupts intestinal barrier function.

**Figure 2:**
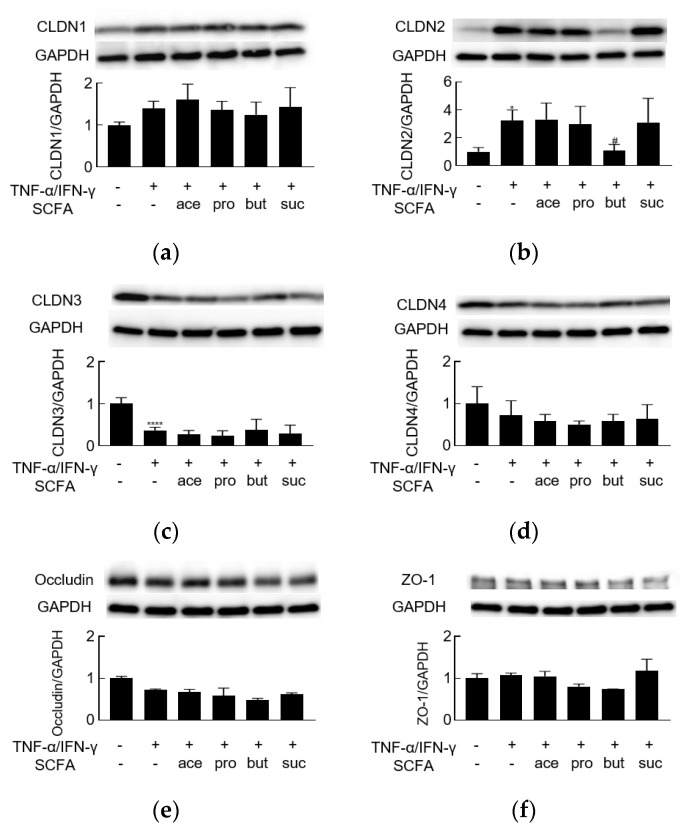
The effect of short-chain fatty acids on TNF-α/IFN-γ-induced dysregulation of tight junction proteins. Western blot analyses of (**a**) claudin-1 (CLDN1, 22 kDa), (**b**) claudin-2 (CLDN2, 22 kDa), (**c**) claudin-3 (CLDN3, 22 kDa), (**d**) claudin-4 (CLDN4, 22 kDa), (**e**) occludin (65 kDa), and (**f**) ZO-1 (220 kDa) proteins were performed following stimulation on the basolateral side of the membrane, with the combination of TNF-α/IFN-γ (10 ng/mL), and on the apical side with acetate (ace), propionate (pro), butyrate (but), or succinate (suc) (2 mM). * *p* < 0.05 compared to medium-treated control; ^#^ *p* < 0.05 compared to TNF-α/IFN-γ group; **** *p* < 0.0001 compared to medium-treated control.

TJs are closely related to barrier function; therefore, we employed Western blotting to investigate changes in the levels of TJ proteins after 24 h of stimulation. Exposure to TNF-α/IFN-γ resulted in significant increases in the level of claudin-2 and decreases in the level of claudin-3 (Figure 2b,c, Supplementary Figure S1). The levels of claudin-4 and occludin were decreased nominally (but not significantly) (Figure 2d,e), while the levels of claudin-1 and ZO-1 did not appear to be altered by TNF-α/IFN-γ stimulation (Figure 2a,f). These data indicated that TNF-α/IFN-γ might disrupt barrier function by modulating the levels of TJ proteins.

### 3.2. Butyrate Alleviates TNF-α/IFN-γ-Induced Barrier Dysfunction

To assess the effect of individual SCFAs on barrier dysfunction induced by TNF-α/IFN-γ, acetate, propionate, butyrate, or succinate (each at 2 mM) was added to the apical side of monolayers at the same time as TNF-α/IFN-γ stimulation was initiated. The TNF-α/IFN-γ-induced decrease in TEER was counteracted by butyrate but not by any of the other SCFAs, even in different concentrations (Figure 1a, Supplementary Figure S2a–c). Furthermore, the effect of butyrate was concentration-dependent (Figure 1b). Regarding the levels of TJ proteins, butyrate (not any of the other SCFAs) attenuated the upregulation of claudin-2 (Figure 2b). However, butyrate did not counteract the TNF-α/IFN-γ-associated depletion of claudin-3 (Figure 2c). Thus, butyrate may alleviate TNF-α/IFN-γ-induced barrier dysfunction via effects on claudin-2 levels.

### 3.3. IL-13 Induces Barrier Dysfunction and Increases the Level of Claudin-2

**Figure 3:**
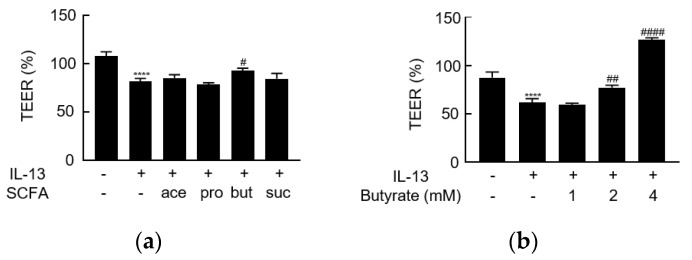
The effect of short-chain fatty acids on IL-13-induced barrier dysfunction. (**a**) TEER values were assessed and are presented as a percentage of control (time point 0) following stimulation on the basolateral side of the membrane with IL-13 (10 ng/mL) and on the apical side with acetate (ace), propionate (pro), butyrate (but), or succinate (suc) (2 mM). (**b**) The concentration-dependent effect of butyrate on IL-13-induced barrier dysfunction. **** *p* < 0.0001 compared to medium-treated control; ^#^ *p* < 0.05 compared to IL-13 group; ^##^ *p* < 0.01 compared to IL-13 group; ^####^ *p* < 0.0001 compared to IL-13 group.

**Figure 4:**
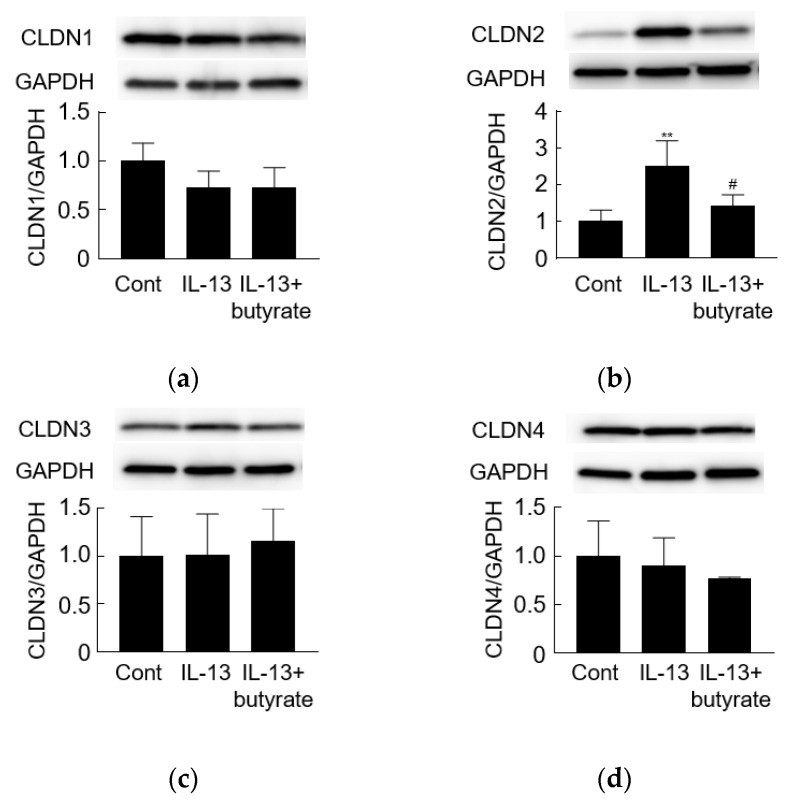

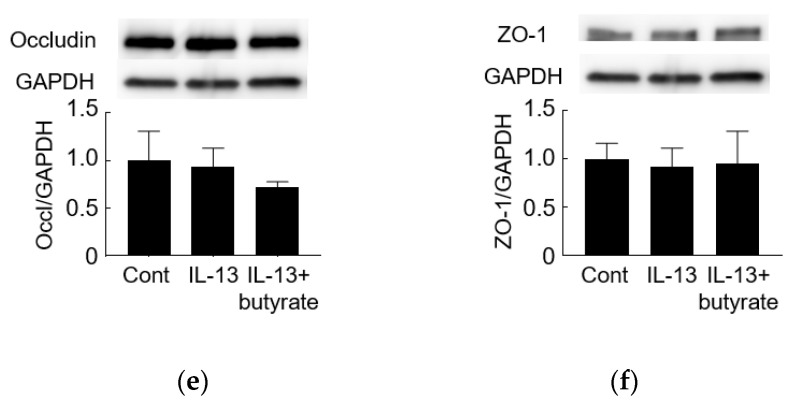
The effect of butyrate on IL-13-induced dysregulation of tight junction proteins. Western blot analyses of (**a**) claudin-1 (CLDN1, 22 kDa), (**b**) claudin-2 (CLDN2, 22 kDa), (**c**) claudin-3 (CLDN3, 22 kDa), (**d**) claudin-4 (CLDN4, 22 kDa), (**e**) occludin (65 kDa), and (**f**) ZO-1 (220 kDa) proteins were performed following stimulation on the basolateral side of the membrane with IL-13 (10 ng/mL) and on the apical side with butyrate (but, 2 mM). ** *p* < 0.01 compared to medium-treated control (Cont); ^#^ *p* < 0.05 compared to IL-13 group.

IL-13 was used to build another pathological model to investigate the effect of butyrate. The addition of IL-13 to the basolateral side of the insert resulted in a significant decrease (compared to the medium-treated control) in TEER after 24 h of stimulation (Figure 3a). IL-13 exposure significantly increased the claudin-2 protein level while resulting in nominal decreases in the level of claudin-1. Levels of claudin-3, claudin-4, occludin, and ZO-1 did not appear to be affected (Figure 4, Supplementary Figure S3).

### 3.4. Butyrate Alleviates IL-13-Induced Barrier Dysfunction

Individual SCFAs were added to the apical side of the membrane, while IL-13 was provided on the basolateral side. Only butyrate ameliorated the IL-13-induced decrease in TEER (Figure 3a, Supplementary Figure S2d–f). Butyrate showed a concentration-dependent effect in this model (Figure 3b). When the effects on TJ protein levels were assessed, butyrate blocked the IL-13-induced upregulation of claudin-2 (Figure 4b) but did not affect the levels of claudin-1, claudin-3, claudin-4, occludin, or ZO-1 (Figure 4). Thus, the effect of butyrate on IL-13-induced barrier function may be mediated via the regulation of claudin-2 levels.

### 3.5. TNF-α/IFN-γ but Not IL-13 Increases Leak Pathway *Permeability*

**Figure 5:**
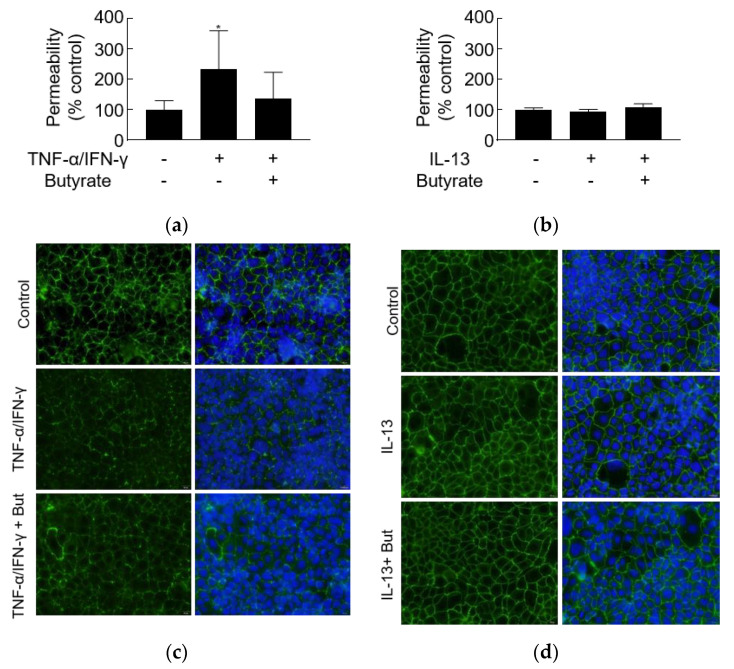
The effect of TNF-α/IFN-γ and IL-13 on leak pathway permeability. FD4 permeability was assessed and is presented as a percentage of control (time point 0) following stimulation on the basolateral side of the membrane with the combination of (**a**) TNF-α/IFN-γ (10 ng/mL) or (**c**) IL-13 (10 ng/mL) and on the apical side with or without butyrate (2 mM). ZO-1 distribution was assessed after stimulation with (**b**) TNF-α/IFN-γ or (**d**) IL-13 with or without butyrate by immunofluorescence staining. * *p* < 0.01 compared to medium-treated control.

Exposure to TNF-α/IFN-γ resulted in significant increases in the permeability of FD4, but butyrate did not significantly relieve the increased leak pathway permeability (Figure 5a). Moreover, TNF-α/IFN-γ caused the dispersion of ZO-1 compared to contiguous ZO-1 staining of control, which might be the reason for increased permeability (Figure 5b). Butyrate appeared to partially alleviate the ZO-1 dispersion caused by TNF-α/IFN-γ (Figure 5b). However, IL-13 did not alter the FD4 permeability and ZO-1 distribution after 24 h of stimulation (Figure 5c,d). These data indicated that the effect of TNF-α/IFN-γ and IL-13 on leak pathway permeability is distinguished.

## 4. Discussion

In the present study, we showed that butyrate, but not other SCFAs, alleviates barrier dysfunction and inhibits the upregulation of claudin-2 induced by TNF-α/IFN-γ and by IL-13. Although previous studies have suggested that each SCFA or the mixture of these molecules have a protective effect on the GI barrier [11,12,13,23], the present work revealed, for the first time (to our knowledge), the effect of each SCFA on the regulation of TJ proteins required for maintenance of the intestinal barrier. Notably, butyrate appears to protect the intestinal barrier through the regulation of claudin-2 levels.

Butyrate has been reported to promote epithelial barrier function via inhibition of histone deacetylases [24], energy supply [25], immune modulation [26], and regulation of TJ [13]. Butyrate’s direct effect has been investigated primarily under normal conditions; fewer studies have evaluated butyrate’s role under pathological conditions [21,27,28]. In the present study, we showed that butyrate counteracts both the TNF-α/IFN-γ- and IL-13-induced upregulation of claudin-2 without affecting the levels of other TJ proteins. These results indicate that claudin-2 is a target, whereby butyrate protects the GI barrier. However, in a previous study, butyrate did not protect against inflammation-induced loss of epithelial barrier function in primary cell monolayers from patients with ulcerative colitis (UC) [29]. The contradictory results of the effect of butyrate might depend on the type of the cultured cells.

Besides, based on the present study, even though TNF-α/IFN-γ and IL-13 uniformly decreased the TEER (pore pathway), their effects were different on FD4 permeability (leak pathway), which is consistent with a previous study [30]. The TNF-α/IFN-γ- and IL-13-induced upregulation of pore pathway permeability could be defined by claudin-2, and the increased permeability of the leak pathway induced by TNF-α/IFN-γ might be associated with the ZO-1 dispersion, which appeared to be relieved by butyrate. The present result indicated that Th1- and Th2-type cytokines [31] might differently affect barrier destruction. However, the present results were partially in contradiction with a previous study [30]. In their study, TNF-α with pretreatment of IFN-γ did not change the level of claudin-2 and ZO-1 distribution. We supposed that the difference might lie in the treatment duration of TNF-α (4 h; present study: 24 h). Other previous reports indicated that TNF-α affected ZO-1 distribution time-dependently [32,33].

Among TJ proteins, claudin-2 has the unique property of serving as a pore-forming TJ protein; the claudin-2 channel is permeable to small cations and water. Upregulation of claudin-2 contributes to diarrhea via disruption of the barrier [34,35]. The level of claudin-2 is increased in the intestinal epithelium of patients with IBS with diarrhea (IBS-D) [36,37], UC [38], and celiac disease [39], an observation that may explain why such patients experience diarrhea [40]. In the present study, TNF-α/IFN-γ, IL-13, and butyrate all appeared to contribute to the regulation of claudin-2 levels. These data confirm the key role of claudin-2 in intestinal permeability. Given the amelioration in IBD symptoms associated with the use of butyrate supplements [25], the regulation of claudin-2 levels appears to be crucial to the pathology of this disease.

Butyrate has been postulated to enhance the GI barrier through regulation of the levels of claudin-1, occludin, and/or ZO-1 proteins [16,41], but effects on those proteins were not seen in the present study. These discrepancies might indicate that the effect of butyrate differs between normal and pathological models. Alternatively, the strength of butyrate’s effects may not be sufficient to alter the levels of TJ proteins other than claudin-2 under pathological conditions. Since butyrate contributes to changes in the levels of claudin-2, but not in the levels of other TJ proteins, butyrate may have a palliative effect on any pathological condition associated with claudin-2 upregulation.

The present study did not address the mechanism whereby butyrate regulates claudin-2 upregulation. However, it has been reported that both TNF-α/IFN-γ and butyrate contribute to the modulation of AMP-activated protein kinase, a protein whose activity regulates apical junctions and alters the barrier function of the intestinal epithelium [42,43]. In addition, TNF-α is known to upregulate claudin-2 expression via phosphatidylinositol-3-kinase (PI3K) signaling [44], which other work has shown can be blocked by butyrate [27]. Moreover, the PI3K pathway also can be activated by IL-13 [22]. Alone or together, these pathways may contribute to the mechanism of the observed effects on TJ function and GI permeability. However, the relevant molecular mechanisms and signaling pathways are likely to form an intricate web; it remains difficult to explain the role of butyrate in regulating the intestinal barrier via a single pathway.

Some limitations exist in the present study. First, only one cell line was used, and the molecular pathway was not investigated. Second, as these experiments were performed only in vitro, further studies in vivo will be needed to assess the potential effect of butyrate in clinical practice.

## 5. Conclusions

We demonstrated that butyrate, but not other SCFAs, ameliorated cytokine-induced barrier dysfunction via down-regulation of claudin-2 levels. It is hoped that these results will facilitate the development of treatments for diseases related to intestinal barrier impairment.

## Data Availability

Data are contained in supplementary material or are available on request from the corresponding author.

## Supplementary Materials

The following are available online at https://www.mdpi.com/2079-7737/10/3/205/s1, Supplementary Figure S1: Full membranes of Western blotting for Figure 2. Supplementary Figure S2: The effect of different concentrations of acetate, propionate and succinate on TNF-α/IFN-γ- and IL-13-induced barrier dysfunction. Supplementary Figure S3: Full membranes of Western blotting for Figure 4.
