# Supplementary material for: Butyrate Alleviates Cytokine-Induced Barrier Dysfunction by Modifying Claudin-2 Levels
Source: Biology (Basel). 2021 Mar 9;10(3):205. doi: 10.3390/biology10030205 (PMC8000923; doi:10.3390/biology10030205)

Supplementary Figure S1. Full membranes of Western blotting for Figure 2.

Bands (Membranes) in the manuscript

ImageQuant; AS 500 was used to capture chemiluminiscence. Color picture and Monochrome pictuires will be generated automatically. Monochrome pictuires were analysed by ImageJ.

The membrane was cut in blotting, so ponceau staining was also shown here to confirm that pieces are form same membrane.

Lane order:											
Control	TNF-α /IFN-γ 10ng/ml	TNF-α /IFN-γ 10ng/ml+ acetate 2mM	TNF-α /IFN-γ 10ng/ml+ propionat e 2mM	TNF-α /IFN-γ 10ng/ml+ butyrate 2mM	TNF-α /IFN-γ 10ng/ml+ succinat e 2mM	Control	TNF-α /IFN-γ 10ng/ml	TNF-α /IFN-γ 10ng/ml+ acetate 2mM	TNF-α /IFN-γ 10ng/ml+ propionat e 2mM	TNF-α /IFN-γ 10ng/ml+ butyrate 2mM	TNF-α /IFN-γ 10ng/ml+ succinat e 2mM

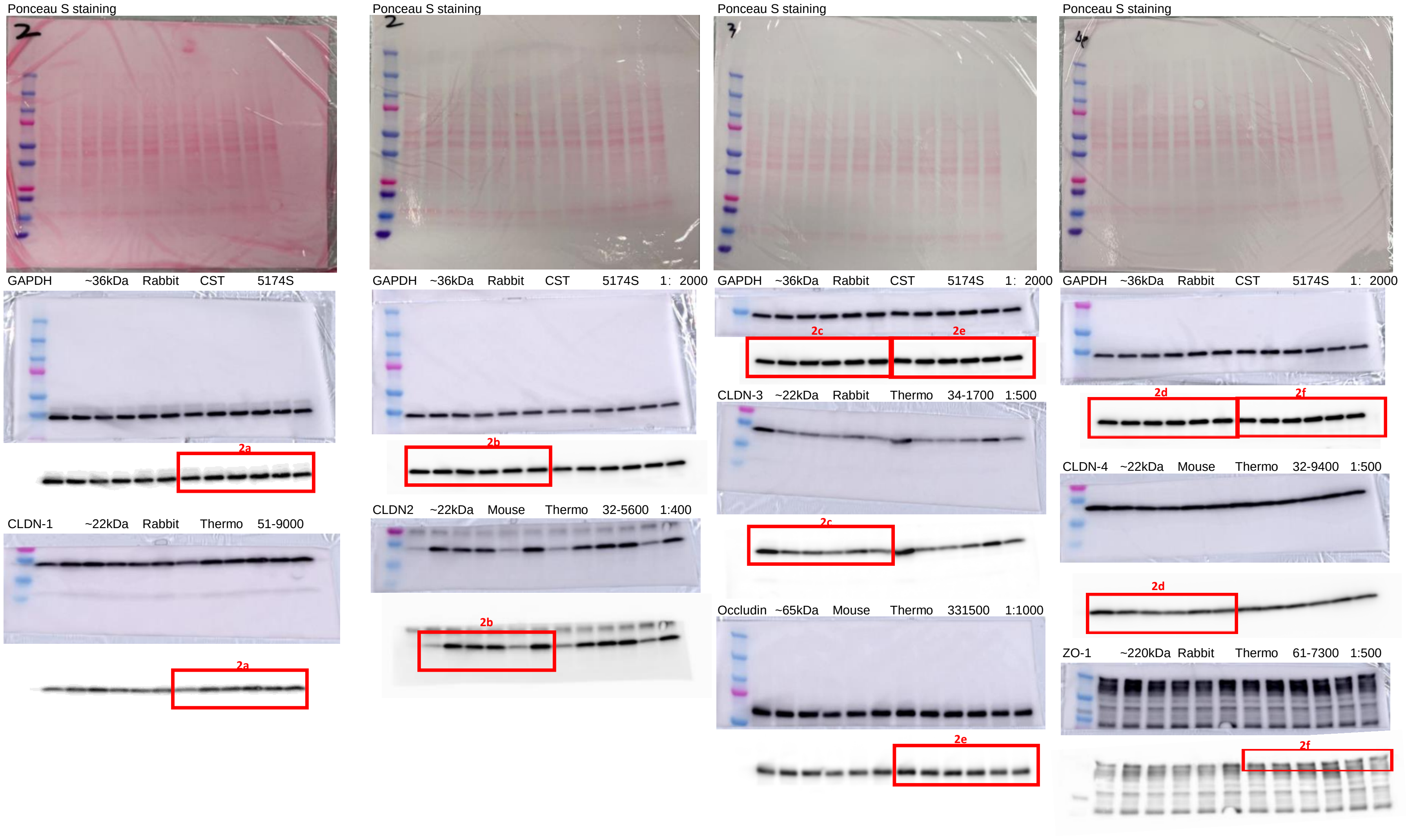

First experiment

Control	TNF-α /IFN-γ 10ng/ml	TNF-α /IFN-γ 10ng/ml+ acetate 2mM	TNF-α /IFN-γ 10ng/ml+ propiona te 2mM	TNF-α /IFN-γ 10ng/ml+ butyrate 2mM	TNF-α /IFN-γ 10ng/ml+ succinat e 2mM	Control	TNF-α /IFN-γ 10ng/ml	TNF-α /IFN-γ 10ng/ml+ acetate 2mM	TNF-α /IFN-γ 10ng/ml+ propiona te 2mM	TNF-α /IFN-γ 10ng/ml+ butyrate 2mM	TNF-α /IFN-γ 10ng/ml+ succinat e 2mM

The membrane was cut in blotting, so ponceau staining was also shown here to confirm that pieces are form same membrane.

Ponceau S staining

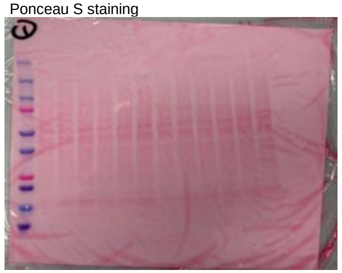

GAPDH ~36kDa Rabbit CST 5174S 1: 2000

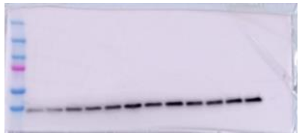

CLDN-4 ~22kDa Mouse Thermo 32-9400 1:500

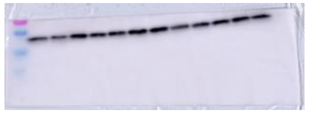

Ponceau S staining

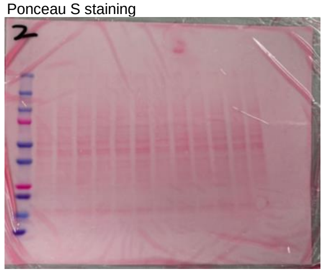

GAPDH ~36kDa Rabbit CST 5174S 1: 2000

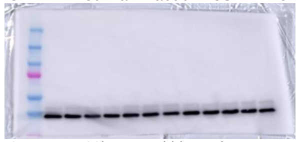

CLDN-1 ~22kDa Rabbit Thermo 51-9000 1:250

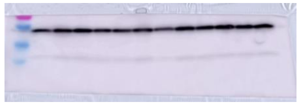

Ponceau S staining

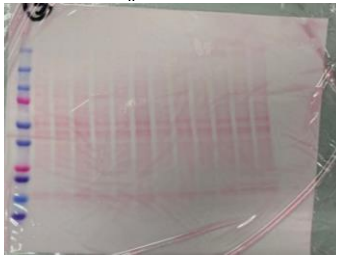

GAPDH ~36kDa Rabbit CST 5174S 1: 2000

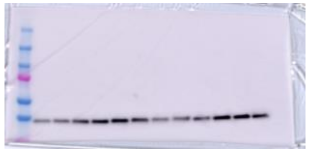

CLDN-3 ~22kDa Rabbit Thermo 34-1700 1:500

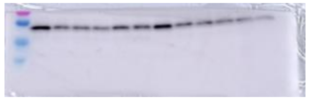

Ponceau S staining

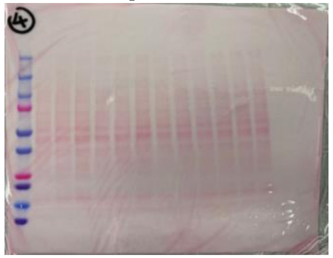

GAPDH ~36kDa Rabbit CST 5174S 1: 2000

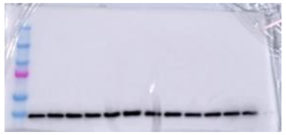

CLDN2 ~22kDa Mouse Thermo 32-5600 1:400

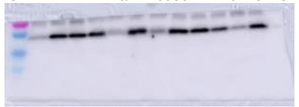

5

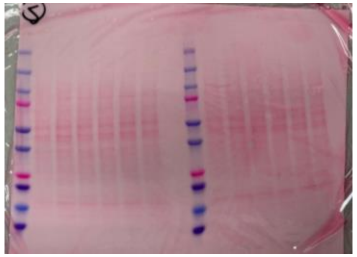

GAPDH ~36kDa Rabbit CST 5174S 1: 2000

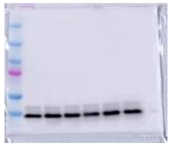

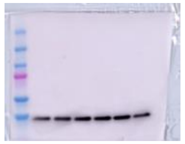

CLDN-1 ~22kDa Rabbit

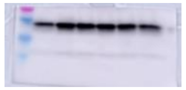

CLDN-4 ~22kDa Mouse

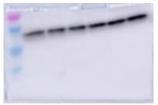

6

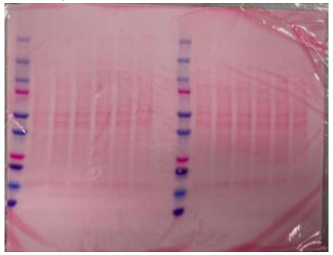

GAPDH ~36kDa Rabbit CST 5174S 1: 2000

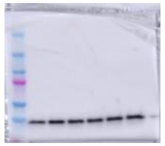

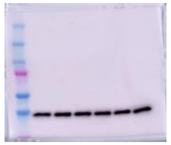

CLDN2 ~22kDa Mouse

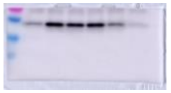

CLDN-3 ~22kDa Rabbit

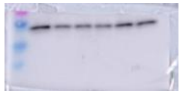

Second repeat

Control	TNF-α /IFN-γ 10ng/ml	TNF-α /IFN-γ 10ng/ml+ acetate 2mM	TNF-α /IFN-γ 10ng/ml+ propionate 2mM	TNF-α /IFN-γ 10ng/ml+ butyrate 2mM	TNF-α /IFN-γ 10ng/ml+ succinate 2mM	Control	TNF-α /IFN-γ 10ng/ml	TNF-α /IFN-γ 10ng/ml+ acetate 2mM	TNF-α /IFN-γ 10ng/ml+ propionate 2mM	TNF-α /IFN-γ 10ng/ml+ butyrate 2mM	TNF-α /IFN-γ 10ng/ml+ succinate 2mM

The membrane was cut in blotting, so ponceau staining was also shown here to confirm that pieces are form same membrane.

Ponceau S staining

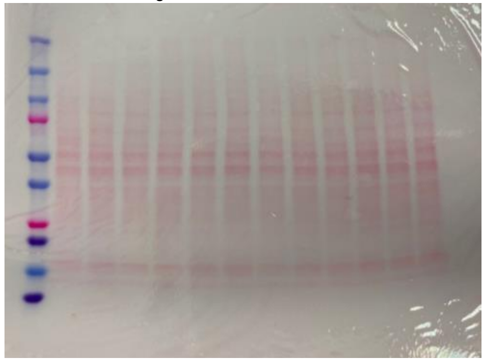

GAPDH ~36kDa Rabbit CST 5174S 1: 2000

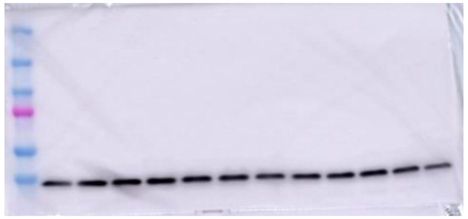

CLDN-1 ~22kDa Rabbit Thermo 51-9000 1:250

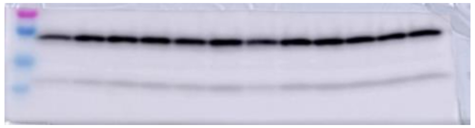

Ponceau S staining

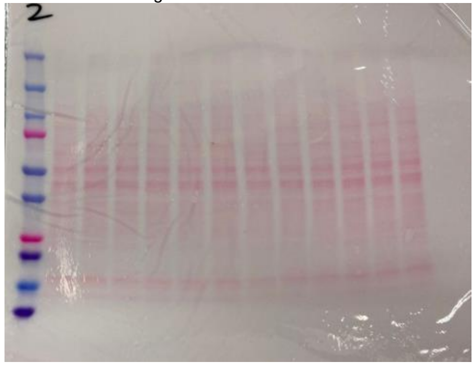

GAPDH ~36kDa Rabbit CST 5174S 1: 2000

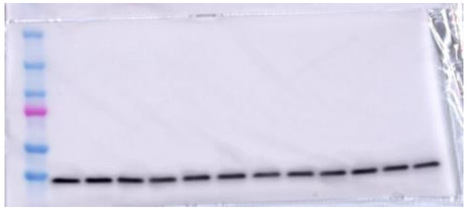

CLDN2 ~22kDa Mouse Thermo 32-5600 1:400

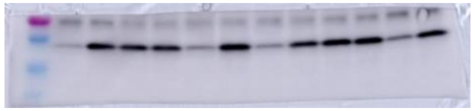

Ponceau S staining

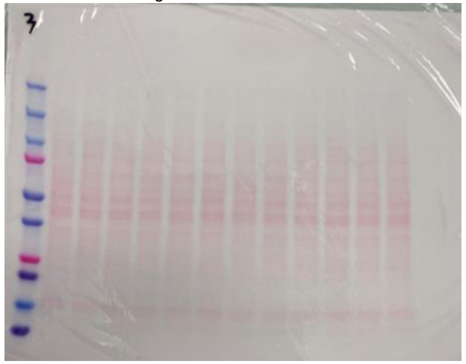

GAPDH ~36kDa Rabbit CST 5174S 1: 2000

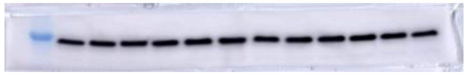

CLDN-3 ~22kDa Rabbit Thermo 34-1700 1:500

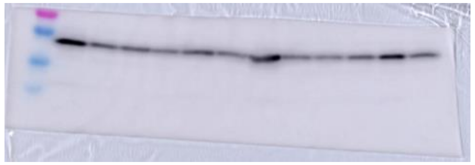

Occludin ~65kDa Mouse Thermo 331500 1:1000

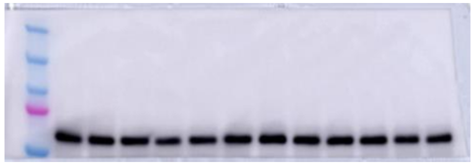

Ponceau S staining

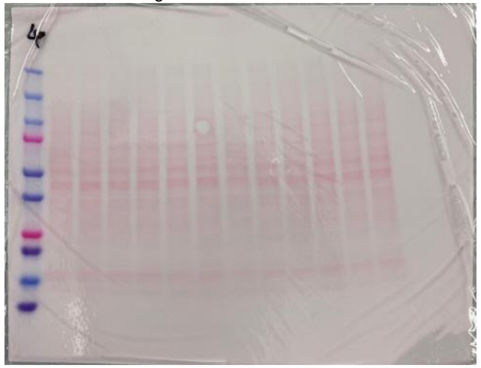

GAPDH ~36kDa Rabbit CST 5174S 1: 2000

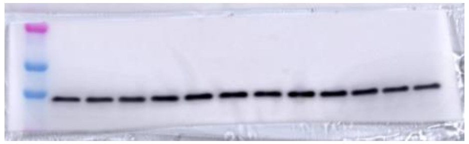

CLDN-4 ~22kDa Mouse Thermo 32-9400 1:500

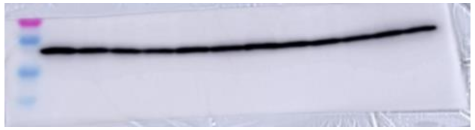

ZO-1 ~220kDa Rabbit Thermo 61-7300 1:500

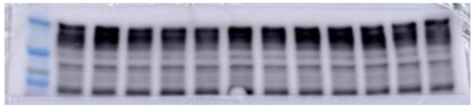

Third repeat

Control	TNF-α /IFN-γ 10ng/ml	TNF-α /IFN-γ 10ng/ml+ butyrate 2mM	Control	TNF-α /IFN-γ 10ng/ml	TNF-α /IFN-γ 10ng/ml+ butyrate 2mM	PC1	PC2

The membrane was cut in blotting, so ponceau staining was also shown here to confirm that pieces are form same membrane.

Ponceau S staining

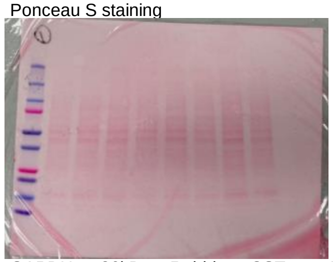

GAPDH ~36kDa Rabbit CST 5174S 1： 2000

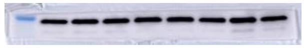

CLDN-1 ~22kDa Rabbit Thermo 51-9000 1:250

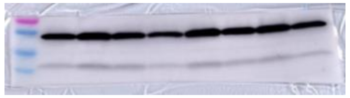

Ponceau S staining

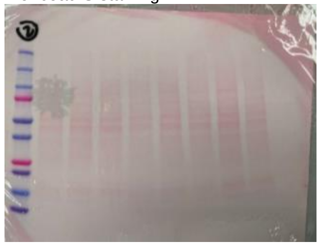

GAPDH ~36kDa Rabbit CST 5174S 1： 2000

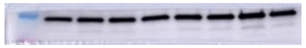

CLDN2 ~22kDa Mouse Thermo 32-5600 1:250

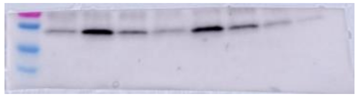

Ponceau S staining

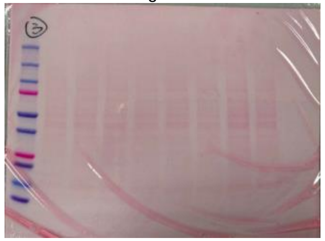

GAPDH ~36kDa Rabbit CST 5174S 1： 2000

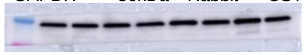

ZO-1 ~220kDa Rabbit Thermo 61-7300 1:500

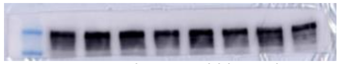

CLDN-3 ~22kDa Rabbit Thermo 34-1700 1:500

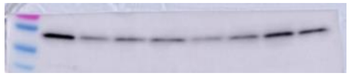

CLDN-4 ~22kDa Mouse Thermo 32-9400 1:500

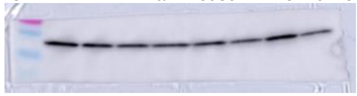

Ponceau S staining

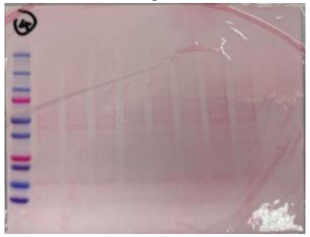

GAPDH ~36kDa Rabbit CST 5174S 1： 2000

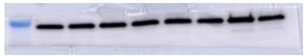

Occludin ~65kDa Mouse Thermo 331500 1:1000

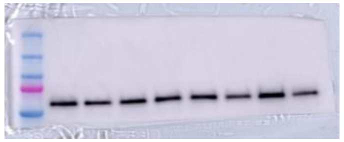

Supplementary Figure S2. The effect of different concentrations of acetate, propionate and succinate on TNF-α/IFN-γ- and IL-13-induced barrier dysfunction. **** $p < 0.0001$ compared to medium-treated control.

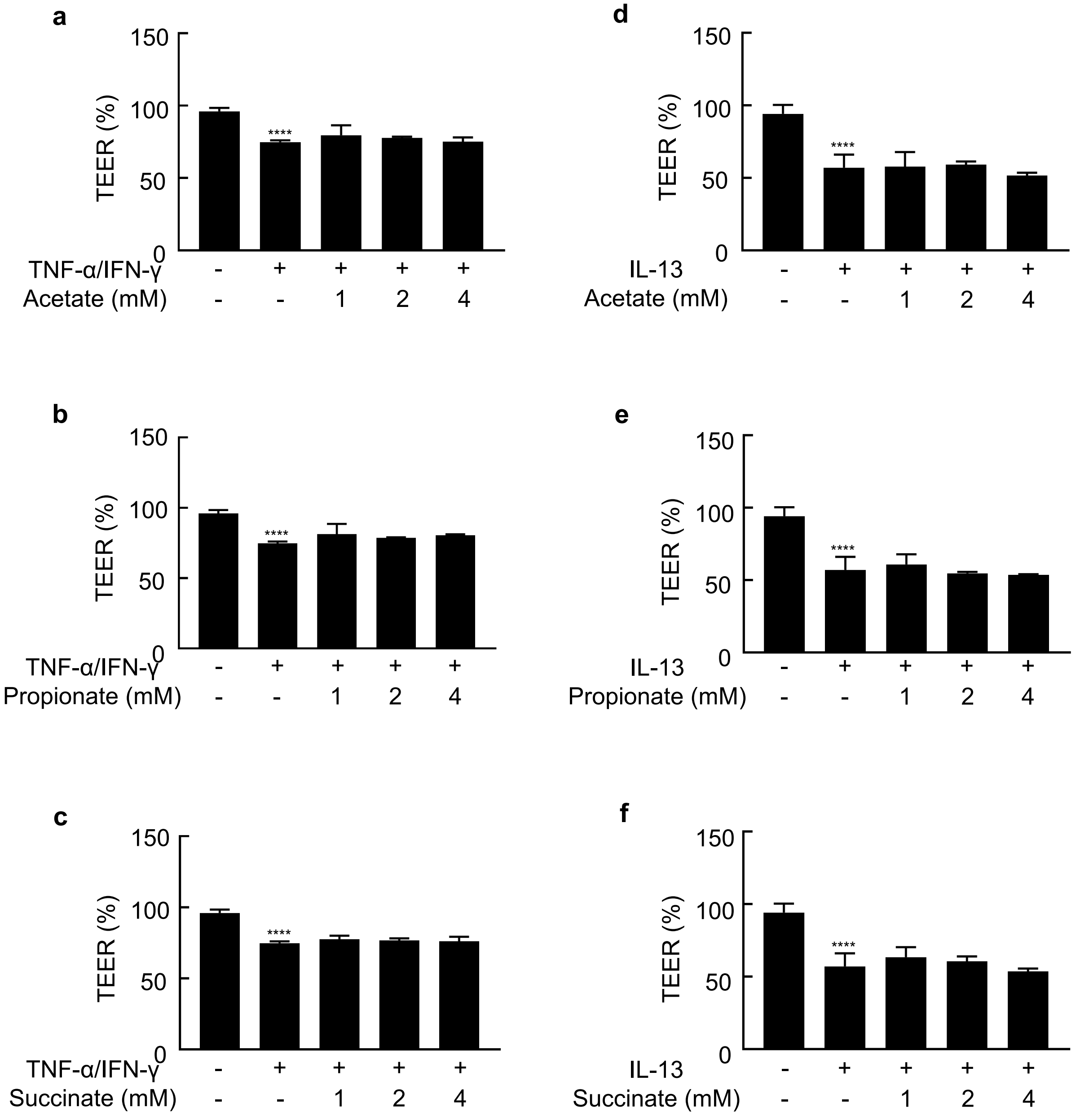

Supplementary Figure S3. Full membranes of Western blotting for Figure 4.

Bands (Membranes) in the manuscript

ImageQuant; AS 500 was used to capture chemiluminiscence. Color picture and Monochrome pictuires will be generated automatically. Monochrome pictuires were analysed by ImageJ.

The membrane was cut in blooting, so ponceau staining was also shown here to confirm that pieces are form same membrane.

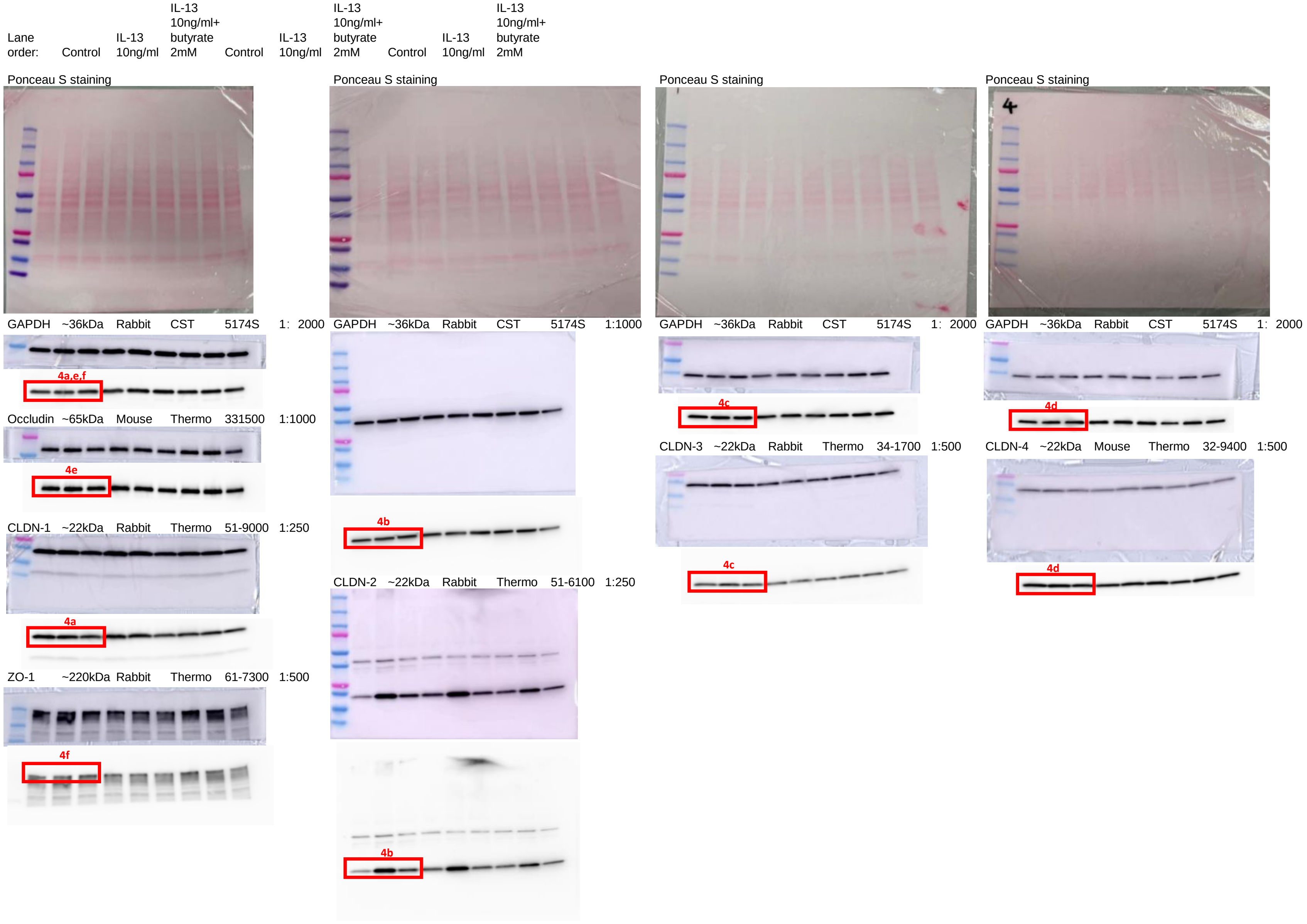

First experiment

Lane order:	Control	IL-13 10ng/ml	IL-13 10ng/ml+ butyrate 2mM	Control	IL-13 10ng/ml	IL-13 10ng/ml+ butyrate 2mM	Control	IL-13 10ng/ml	IL-13 10ng/ml+ butyrate 2mM

Ponceau S

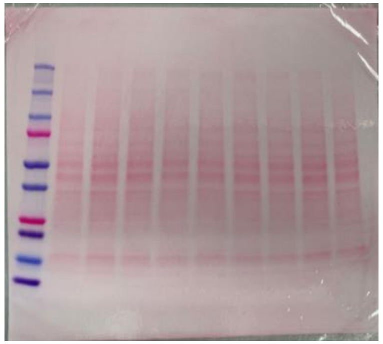

Occludin ~65kDa Mouse Thermo 331500 1:1000

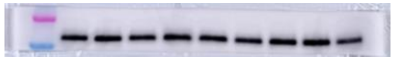

CLDN-1 ~22kDa Rabbit Thermo 51-9000 1:250

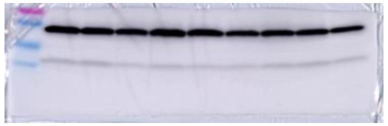

ZO-1 ~220kDa Rabbit Thermo 61-7300 1:500

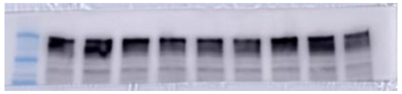

GAPDH ~36kDa Rabbit CST 5174S 1: 2000

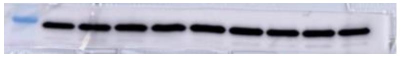

Ponceau S staining

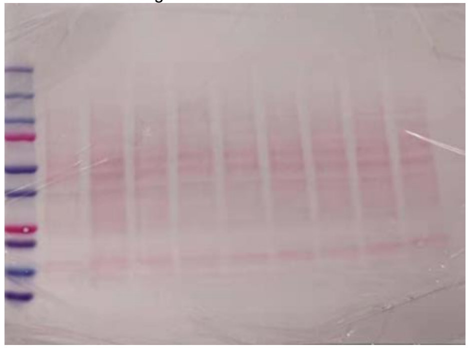

CLDN-2 ~22kDa Rabbit Thermo 51-6100 1:250

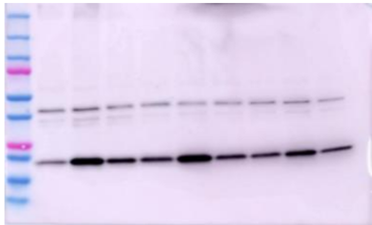

GAPDH ~36kDa Rabbit CST 5174S 1:1000

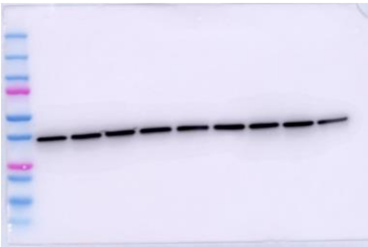

Ponceau S staining

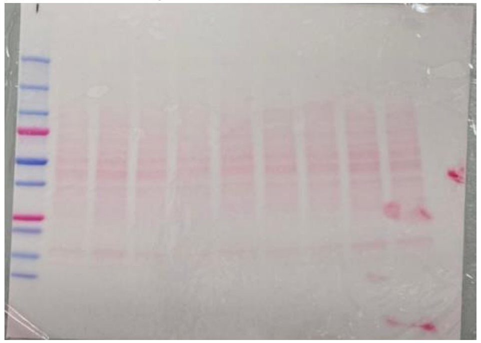

GAPDH ~36kDa Rabbit CST 5174S 1: 2000

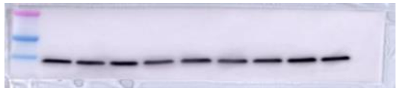

CLDN-3 ~22kDa Rabbit Thermo 34-1700 1:500

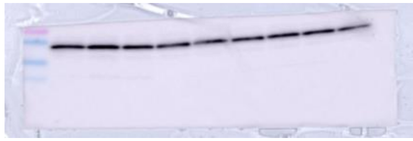

GAPDH ~36kDa Rabbit CST 5174S 1: 2000

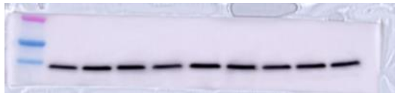

CLDN-4 ~22kDa Mouse Thermo 32-9400 1:500

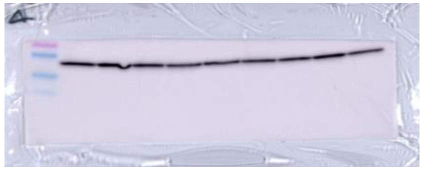

Second repeat

Lane order:									
	Control	IL-13 10ng/ml	IL-13 10ng/ml+ butyrate 2mM	Control	IL-13 10ng/ml	IL-13 10ng/ml+ butyrate 2mM	Control	IL-13 10ng/ml	IL-13 10ng/ml+ butyrate 2mM

Ponceau S staining

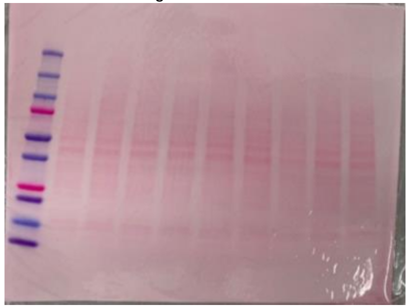

CLDN-1 ~22kDa Rabbit Thermo 51-9000 1:250

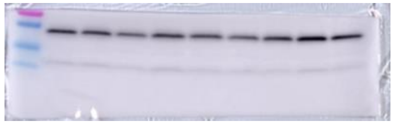

GAPDH ~36kDa Rabbit CST 5174S 1: 2000

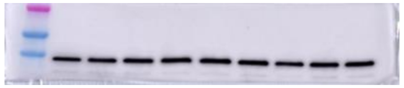

Ponceau S staining

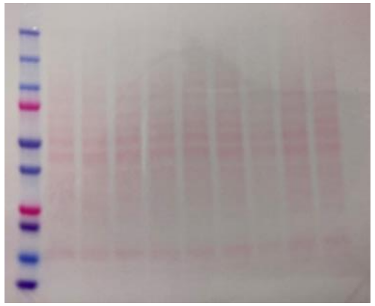

CLDN-2 ~22kDa Rabbit Thermo 51-6100 1:250

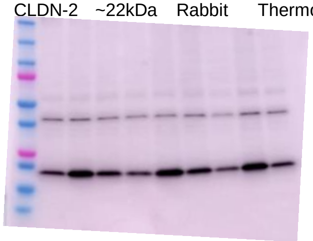

GAPDH ~36kDa Rabbit CST 5174S 1:1000

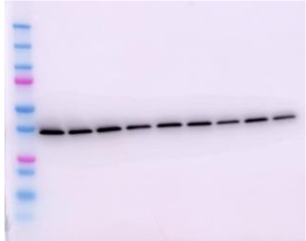

Occludin ~65kDa Mouse Thermo 331500 1:1000

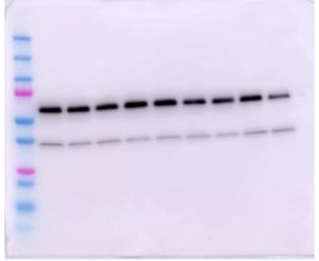

Ponceau S staining

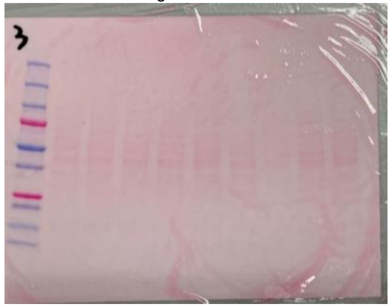

GAPDH ~36kDa Rabbit CST 5174S 1: 2000

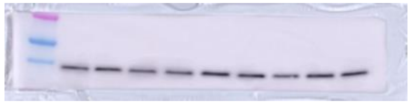

CLDN-3 ~22kDa Rabbit Thermo 34-1700 1:500

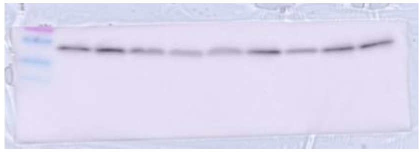

Ponceau S staining

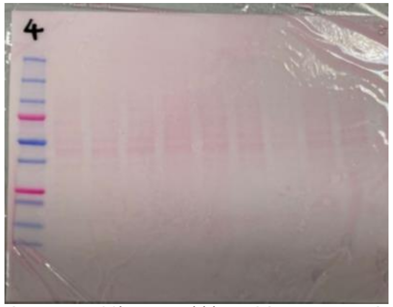

GAPDH ~36kDa Rabbit CST 5174S 1: 2000

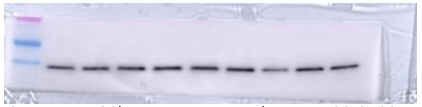

CLDN-4 ~22kDa Mouse Thermo 32-9400 1:500

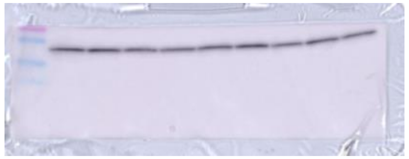

Third repeat

Lane order:	Positive control	Control	IL-13 10ng/ml	IL-13 10ng/ml+ acetate 2mM	IL-13 10ng/ml+ propionate 2mM	IL-13 10ng/ml+ butyrate 2mM	IL-13 10ng/ml+ succinate 2mM	Positive control	Control	IL-13 10ng/ml	IL-13 10ng/ml+ acetate 2mM	IL-13 10ng/ml+ propionate 2mM	IL-13 10ng/ml+ butyrate 2mM	IL-13 10ng/ml+ succinate 2mM

Ponceau S staining

1--1

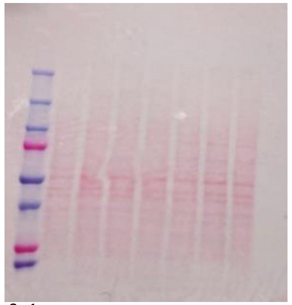

1--2

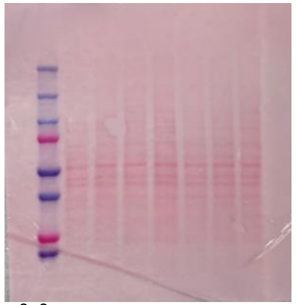

2--1

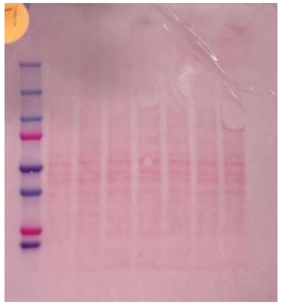

2--2

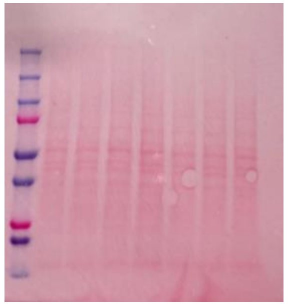

CLDN-1 ~22kDa Rabbit Thermo 51-9000 1:250

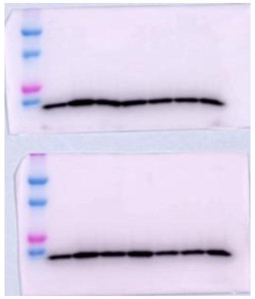

GAPDH ~36kDa Rabbit CST 5174S 1:2000

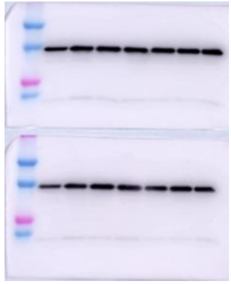

ZO-1 ~220kDa Mouse Thermo 339100 1:500

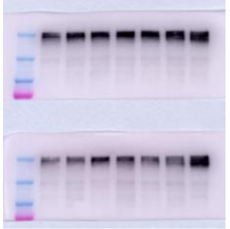

CLDN-2 ~22kDa Rabbit Thermo 51-6100 1:250

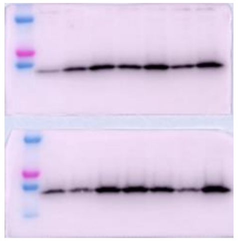

GAPDH ~36kDa Rabbit CST 5174S 1:2000

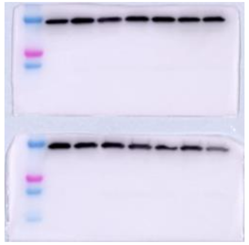

Occludin ~65kDa Mouse Thermo 331500 1:1000

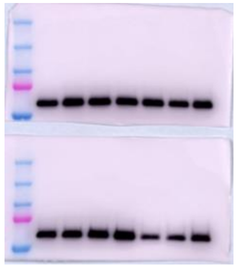

Supplement: Supplementary file 1 [file biology-10-00205-s001.pdf]
